# Abdominal Pain Caused by Occlusion of the Celiac Trunk and Superior Mesenteric Artery in Addition to Irritable Bowel Syndrome: Case Series and Literature Review

**DOI:** 10.7759/cureus.15729

**Published:** 2021-06-17

**Authors:** Zeenat Khuda Bakhsh, Raheel Khan, Khalid Bashir

**Affiliations:** 1 Emergency Medicine, Hamad Medical Corporation, Doha, QAT; 2 Medicine, Qatar University, Doha, QAT; 3 Emergency Medicine, Weill Cornell Medical College - Qatar, Doha, QAT

**Keywords:** irritable bowel syndrome, sma occlusion, abdominal pain, mesenteric ischemia, vascular thrombosis, splenic infarcts

## Abstract

Irritable bowel syndrome (IBS) is a benign condition of the gastrointestinal tract causing abdominal pain, bloating, diarrhea, and/or constipation. Symptoms of IBS usually improve on passing flatus and defecation. There is no known identifiable underlying pathology; however, several risk factors are known to contribute to the development of IBS, which include a stressful lifestyle and certain foods such as bread, coffee, alcohol, pasta, and chocolates. Intestinal bacteria may also contribute to symptoms of IBS. IBS is diagnosed clinically and treated with various medications to control the symptoms. On the other hand, celiac and mesenteric artery thrombosis (CAMAT) is a condition that may cause significantly higher mortality and morbidity if not recognized early. CAMAT leads to the blockage of major blood vessels to the intestine and several abdominal viscera leading to abdominal pain, nausea, sweating, and, in some cases, symptoms of shock. CAMAT is most likely caused by thrombosis; however, occasionally, embolisms from distant sources in patients with atrial fibrillation can also contribute to the development of CAMAT. CAMAT is usually diagnosed with a computed tomography angiogram (CTA) and treated either surgically or medically with anticoagulants. Vascular thrombus in the thoracic and abdominal region causing ischemia of the stomach and abdominal pain in patients with a history of IBS can easily be missed and cause grave complications with high morbidity and mortality. We present two cases who were initially diagnosed and treated for IBS and later diagnosed with serious intra-abdominal pathology of CAMAT thrombosis.

The first case is of a 55-year-old female who was previously diagnosed with IBS and was treated with mebeverine 200mg twice daily and esomeprazole 20mg once daily for 10 weeks. Her pain continued to get worse and she presented to the emergency department by ambulance. She underwent CTA, which showed occlusion of the celiac trunk and superior mesenteric artery causing liver and splenic infarcts. The patient received heparin and underwent a thrombectomy and embolectomy of the superior mesenteric and celiac arteries. No significant abnormality was found in the blood results. Thrombophilia screening was negative. The patient was discharged on warfarin. The second case is of a 53-year-old man who was also initially diagnosed with IBS and was treated with mebeverine 200mg twice daily for eight weeks before presenting to the emergency department with worsening abdominal pain. He underwent a CTA with contrast, which showed occlusion of the common hepatic artery and stenosis of the splenic artery leading to multiple splenic infarcts. No significant abnormality was found in blood test. Thrombophilia screening was negative. He was treated with new anticoagulant medication, dabigatran 150 mg orally twice daily.

Both patients were managed with successful outcomes and were discharged home on anticoagulants. There was no recurrence of symptoms at three-month follow-up.

These cases highlight that a secondary cause of symptoms such as vascular thrombosis must be sought for patients who fail to improve with conservative management of IBS.

## Introduction

Irritable bowel syndrome (IBS) is a functional disorder of the gastrointestinal tract causing long-term recurrent abdominal pain. Patients may also suffer from a bloating sensation with excessive flatus, diarrhea, or constipation. The diagnosis of IBS is based on the Rome IV criteria [1]. Patients with IBS usually present with pain and abdominal discomfort, which often improves on passing flatus and by defecation. There is no known cause of IBS; however, overactivity of the nerves and muscles due to underlying stressful life events, intolerance to certain foods, and certain stomach bacteria may contribute to its development. IBS is clinically diagnosed, and symptoms are treated with antispasmodic medications such as mebeverine (smooth muscle relaxant). The patients are recommended to exercise regularly, manage stress levels, and take medications to provide symptomatic relief.

Thoraco-abdominal aortic thrombus leading to mesenteric ischemia is a rare presentation in the emergency department. Diagnosis requires a high index of suspicion especially in patients with risk factors, which include cardiac embolus related to arrhythmia or valvular heart disease, peripheral artery disease, vasoconstrictive medications, hemodialysis, hereditary or acquired thrombotic conditions, infections, and hypovolemia [2]. Delays in diagnosis and treatment of thrombi lead to high mortality [3]. Patients with mesenteric ischemia typically present with abdominal pain out of proportion on physical examination. Other rare symptoms include nausea, vomiting, diarrhea, rectal bleeding, and/or hypotension. Early diagnosis and management of mesenteric artery thrombosis results in better prognosis. There is a 50% chance of survival if a diagnosis is made within 24 hours of presentation and decreases by 30% if the diagnosis is made beyond 24 hours [1]. Blood investigation may show metabolic acidosis, high lactic acid levels, or increased leukocyte count. X-ray of the abdomen may not reveal any abnormality; therefore, a computed tomography angiogram (CTA) is the choice of investigation for this condition. Mesenteric resonance angiography (MRA) is an alternative in patients in whom CT contrasts cannot be administered. Treatment in the emergency department starts with analgesics (narcotics) and fluid resuscitation to correct hypotension and metabolic abnormalities. Anticoagulation therapy with heparin is started intravenously. If the patient develops signs of peritonitis, emergency surgery should be considered. If the patient presents without signs of bowel infarction, an endoscopic removal of thrombus or thrombolysis may be considered. Thrombolysis is performed by placement of an infusion catheter in the region of the thrombosis followed by infusion of a tissue plasminogen activator (tPA).

## Case presentation

Case 1

**Figure 1:**
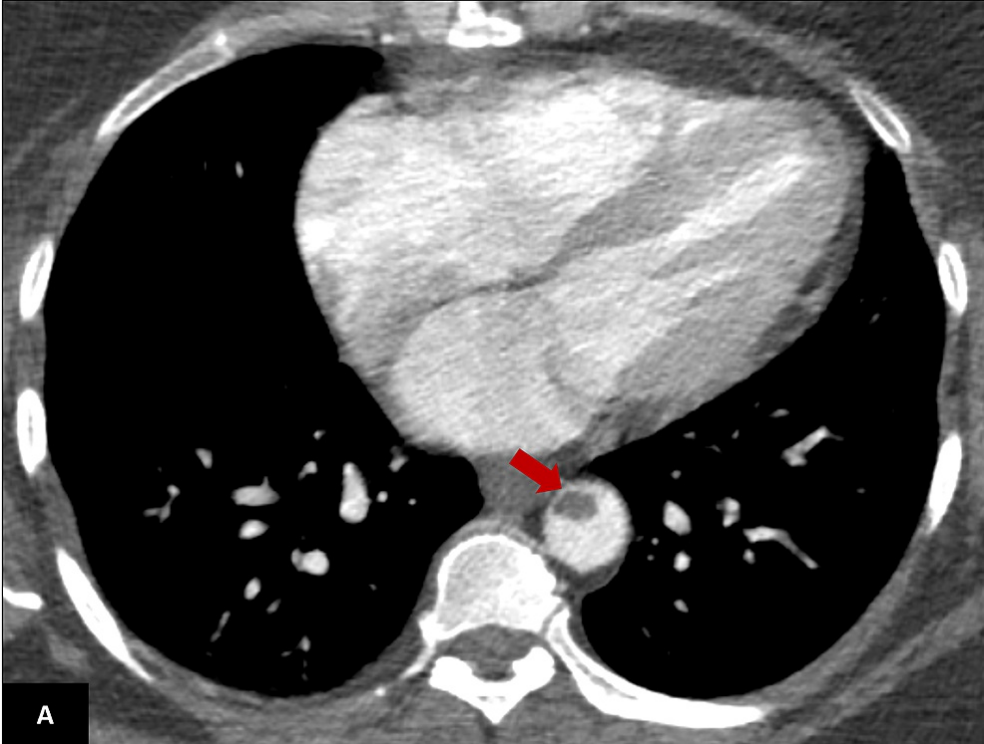
Axial contrast-enhanced CT scan of the lower thorax demonstrating a filling defect within the lower thoracic aorta representing a thrombus (red arrow).

**Figure 2:**
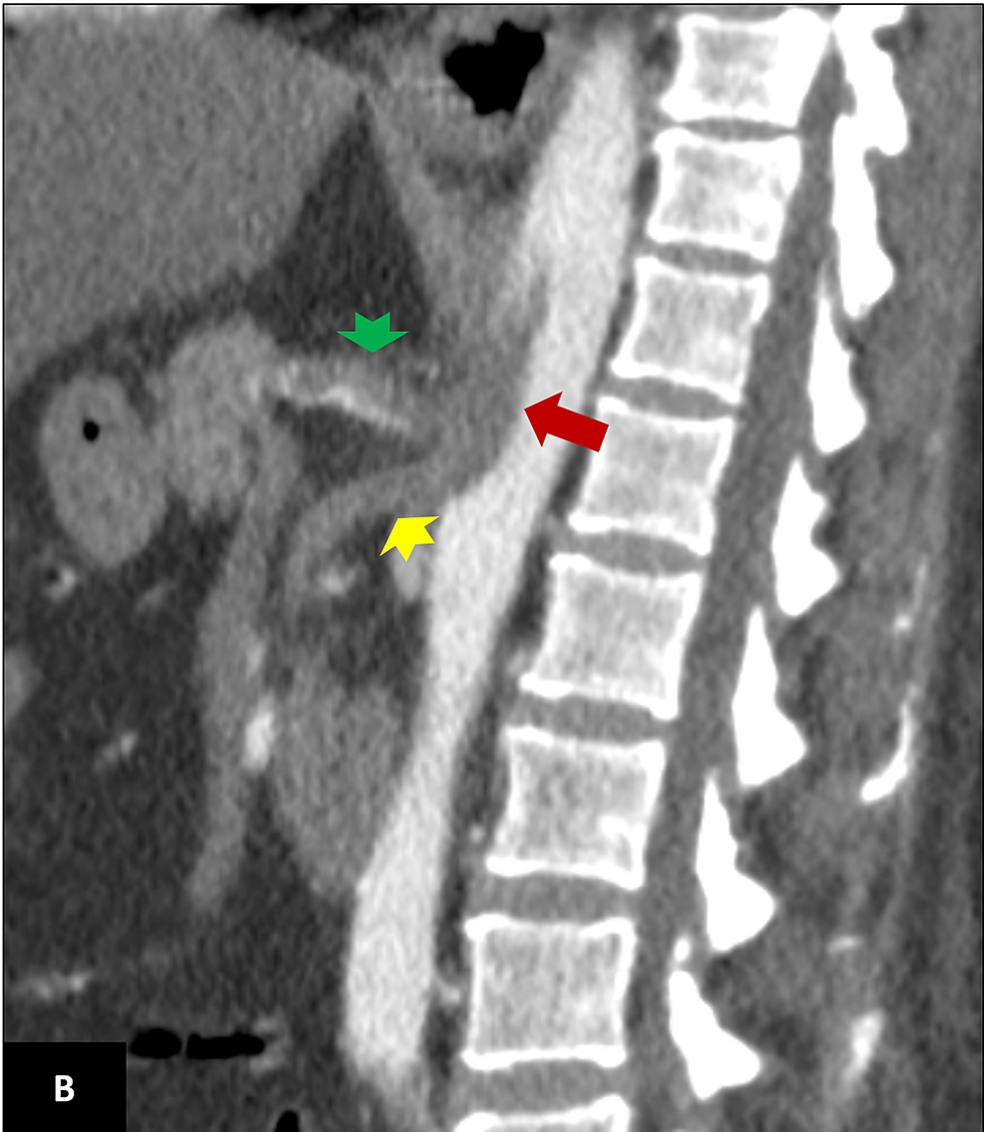
Sagittal contrast-enhanced CT scan of the same patient showing a large thrombus (red arrow) extending from the abdominal aorta and almost completely occluding the celiac trunk (green arrow head) and the superior mesenteric artery (yellow arrow head).

A 55-year-old female with a history of IBS and reflux disease presented to the emergency department with severe abdominal pain radiating to the left hypochondrium and back associated with multiple episodes of vomiting. The patient had stable vital signs. Physical examination showed left upper quadrant tenderness with no guarding. Initial laboratory investigations showed a slight increase in liver enzymes. An ultrasound was performed, which was unremarkable. Due to persistent pain, a CTA was performed. The CTA showed an intramural thrombus of the descending aorta (Figure 1) at the level of the superior mesenteric artery leading to complete occlusion of the celiac tract with complete obstruction of the splenic artery and a part of the hepatic artery causing partial liver infracts and multiple splenic infracts (Figure 2).

The patient was started on a heparin infusion, and vascular surgeons were consulted. The patient underwent thrombectomy and embolectomy of the superior mesenteric and celiac arteries on the same day. The patient’s post-operative course was unremarkable. Thrombophilia screening was negative. The patient was started on warfarin and was discharged in good general condition.

Case 2

**Figure 3:**
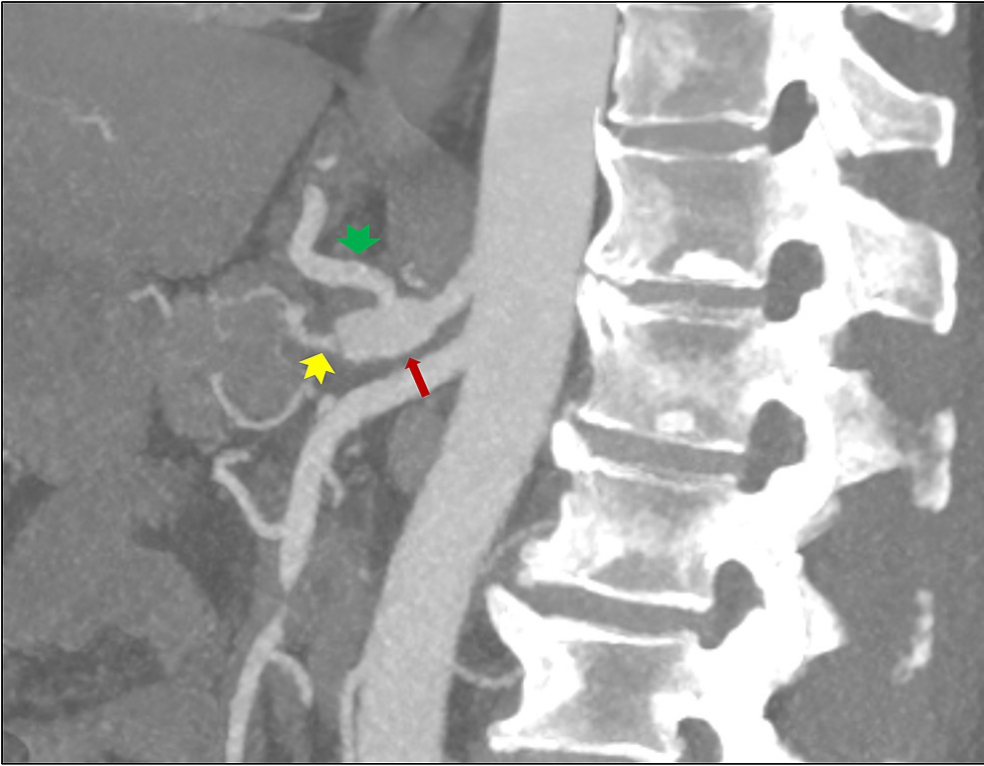
Sagittal maximal intensity projection showing focal dilatation of the celiac trunk (red arrow), patent and prominent left gastric artery (green arrow head), and patent and thick-walled splenic artery (yellow arrow head).

**Figure 4:**
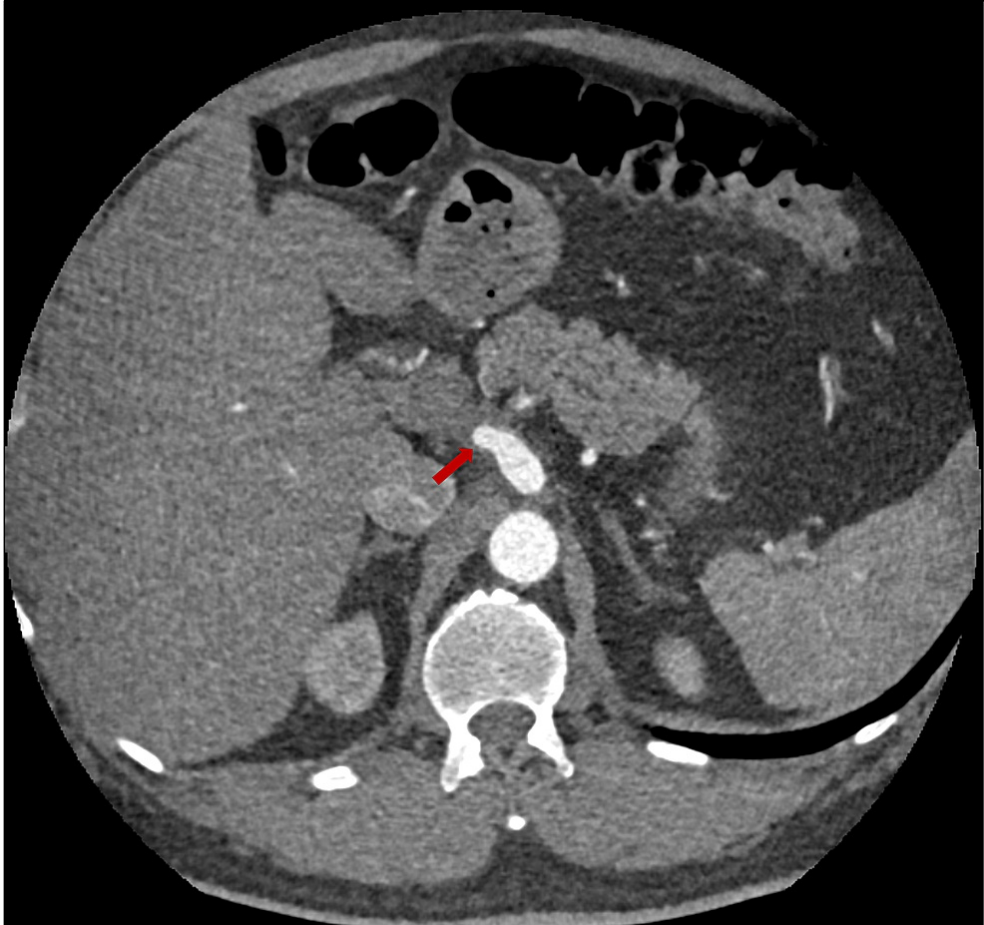
Axial contrast-enhanced CT scan of the abdomen showing abrupt cutoff at the common hepatic artery (red arrow).

**Figure 5:**
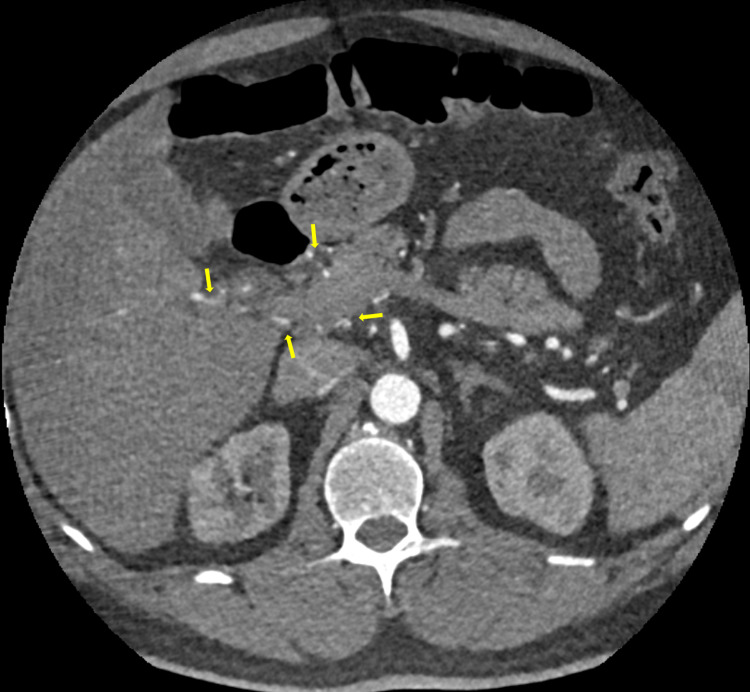
Multiple collaterals at the porta-hepatis and the peri-pancreatic regions (yellow arrows).

A 53-year-old man was initially diagnosed with IBS and treated with mebeverine 200mg twice daily for several weeks before presenting to the emergency department with worsening abdominal pain. The abdomen was soft and lax with mild tenderness. The bowel sounds were normal. The patient underwent CT with contrast, which revealed dilation of the celiac trunk (Figure 3) with occlusion of the common hepatic artery and stenosis of the splenic artery leading to multiple splenic infarcts (Figures 4, 5). The patient was resuscitated with fluids, appropriate analgesia, and anticoagulants. Intravenous heparin was started. The patient was discharged after one week following the initial presentation with oral anticoagulants. Three months post-discharge, the patient remained asymptomatic.

 

## Discussion

IBS is a common cause of functional disorder of the gastrointestinal tract, presenting as abdominal pain and alteration of bowel habit. Its prevalence ranges from 9% to 23% of the worldwide population [4]. The mechanism of the disease is poorly understood but linked to psychosocial factors and changes in stomach physiology. IBS is diagnosed on the basis of the Rome IV criteria. It can be debilitating in some patients, while some patients may have mild-to-moderate symptoms [5]. Before making the diagnosis, it is crucial to rule out organic gastrointestinal disorders that may present as IBS especially in patients with concerning features that include presenting symptoms after the age of 50 years, unexplained weight loss, family history of organic gastrointestinal disorders, gastrointestinal blood loss, and unexplained iron deficiency or anemia. Both of the patients in this case study were initially diagnosed and treated for IBS.

Acute celiac and mesenteric occlusion due to intramural aortic thrombus causing ischemia is a life-threatening cause of abdominal pain in patients presenting to the emergency department [6]. Diagnosis is challenging, and delays in diagnosis carry high mortality. Etiologic factors include atherosclerosis, thrombophilia such as thrombocytosis, protein S, protein C, antithrombin III deficiency, and malignancies [7]. Patients mostly present with nonspecific abdominal pain, which makes it difficult to differentiate it from benign conditions especially in hemodynamically stable patients. CT with contrast is the best diagnostic modality as it is not time-consuming and readily available in most emergency departments [8].

Treatment options include surgery and endovascular thrombolysis or both depending on the hospital resources. Studies show that endovascular treatment has the same efficacy as a surgical approach, with no significant mortality differences between the two methods [9-10].

Both cases highlighted that if abdominal pain symptoms get worse in patients with previously diagnosed IBS, an alternative and more serious pathology should be considered. Early diagnosis and treatment prevent long-term complications in these patients. Both patients presented to the hospital through emergency medical services. Early diagnosis and treatment resulted in prevention of complications.

Vascular thrombosis causing visceral ischemia is a challenging diagnosis because it is a rare entity and requires a high index of suspicion to test, particularly in patients with a secondary diagnosis such as functional gut disorder. We should consider it as part of differentials in patients presenting to the emergency department with disproportionate abdominal pain.

## Conclusions

It is crucial in the emergency department to rule out serious and life-threatening causes of abdominal pain especially in patients with risk factors. Vascular thrombosis causing visceral ischemia is challenging to diagnose as it is rare and needs a high index of suspicion, particularly in patients with functional gut disorder. We should consider it as part of differentials in patients presenting to emergency department with disproportionate abdominal pain.
